# PROMs: Patient related or patient centred? A bridge between Patient-Reported Outcome Measures (PROMs) and clinical ethics in the context of a cohort of gynaeco-oncological patients analysed by qualitative semi- structured interviews

**DOI:** 10.1186/s41687-026-01050-z

**Published:** 2026-03-28

**Authors:** Susanne Theis, Hamideh Fruehwein, Annette Hasenburg, Markus Moehler, Norbert W. Paul

**Affiliations:** 1 Department of Gynaecology and Obstetrics, Johannes Gutenberg University Mainz, Mainz, Germany; 2 Institute for History, Theory and Medical Ethics, Johannes Gutenberg University Mainz, Mainz, Germany; 3 Department of Internal Medicine, Johannes Gutenberg University Mainz, Mainz, Germany

**Keywords:** Proms, Gynaecologic cancer, Qualitative research, Semi-structured interviews, Grounded theory

## Abstract

**Background:**

Patient-reported outcome measures (PROMs) are widely used in oncologic care, yet it remains unclear to what extent they capture the depth and contextual meaning of patient experience in gynecologic oncology.

**Objective:**

To explore gynecologic oncology patients’ narratives of treatment experience, needs, and psychosocial impacts, and to examine how these narratives align with domains covered by commonly used PROMs.

**Methods:**

We conducted semi-structured, in-depth interviews with 20 female participants (31–72 years) across different therapy phases. Interviews were analysed using a constructivist grounded theory approach, including iterative inductive coding and constant comparison. After the inductive category structure stabilized, we conducted a secondary mapping of interview-derived categories to domains represented in commonly used PROM instruments in gynecologic and breast oncology.

**Results:**

Participants described treatment effects not only as physical symptoms and functional limitations, but as experiences embedded in identity, autonomy, coping, and relational roles. While PROM domains broadly covered major areas such as physical symptoms, psychological wellbeing, social functioning, body image, and sexual health, the interview narratives repeatedly emphasized meaning, context, and interpersonal dynamics that were insufficiently represented within standardized PROM structures.

**Conclusion:**

PROMs are a valuable component of oncologic care and efficiently capture selected domains of patient experience. However, our findings indicate that routinely used PROMs in gynecologic oncology may not adequately capture the depth, context, and meaning of patients’ narratives, particularly regarding identity, relational dynamics, and coping. Integrating PROMs with qualitative approaches may better support individualized, patient-centred care in the context of personalized and precision oncology.

**Supplementary Information:**

The online version contains supplementary material available at 10.1186/s41687-026-01050-z.

## Introduction

In an era of increasingly specialized and personalized medicine, patients face a range of diagnostic and therapeutic which have to be weighed not only against the background of clinical outcomes but also in the light of individual goals and preferences. Here, shared decision-making becomes both, more complex and more essential. To involve patients as partners in the process of clinical decision-making - including treatment initiation, escalation, de-escalation or palliative transition - becomes an indispensable part of a timely oncological care. Because of high levels of individualization of diagnostic and therapeutic strategies, decisions must be driven not only by biomedical indicators but also by patients’ individual values, goals, and preferences. Notably, this situation requires tools that are both, clinically feasible and ethically robust.

Integration of Patient Reported Outcome Measures (PROMs) in clinical practice is long since a valuable option to capture patients’ perspectives and experiences systematically in participative decision-making [1] . The application of PROMs is well established in research to evaluate impact of treatment, to monitor compliance or quality of life [2]. Nevertheless, there is a discrepancy between results of PROMs and utilisation of findings from these [3, 4]. According to a review from 2020 this was due to a lack of time of health professionals and lack of knowledge to meaningfully interpret and integrate PROM data into their daily practice as well as systemic barriers to integrate PROMs into clinical workflows on the basis of inadequate information technology infrastructures [4, 5]. Despite these practical limitations, their use is increasing in recent years [6].

PROMs are validated instruments with mostly fixed scales. This enables a high degree of standardization and comparability between individuals (groups) or time points. Being highly practical and cost-effective at the same time makes these instruments an important component of quality measurement and the evaluation of therapies in everyday clinical practice in oncology [1]. Simultaneously, this approach entails limitations, including reduced sensitivity to specific contexts, a lack of flexibility, and consequently, the risk of insufficiently capturing subjective experiences and relational dynamics [7]. The growing implementation of electronic PROMs offers significant advantages, such as rapid data availability and the potential for timely clinical intervention. However, it simultaneously carries the risk of further neglecting critical psychosocial and relational dimensions of patient care, particularly as medical staff may develop a false sense of confidence regarding the comprehensiveness of the information provided [8].

Medically speaking, we have achieved a high level of precision medicine [9–15]. The discovery and development of personalized therapeutic approaches based on biomarkers and cellular metabolic processes, combined with rapid technological advances in diagnostic and biomedical imaging, have led to an entirely new form of personalized medicine in recent years [15]. In light of these highly dynamical technological and medical advancements in precision medicine, from an interdisciplinary perspective (health sciences, socially and ethically) it is now essential to critically examine whether the individual behind the patient is considered sufficiently - ensuring that personal needs, values, and concerns are integrated into care in a genuinely person-centred and ethically responsible manner. From a psychological perspective, this entails addressing patients’ fears, emotional distress, and mental health needs [16, 17]. Sociologically, factors such as social inclusion, family dynamics, and cultural background significantly shape patients’ experiences of illness [18]. Ethically, patient-centred care requires respect for individual autonomy and its boundaries as well and personal values - particularly in complex contexts such as cancer diagnosis, end-of-life decision-making, and the equitable allocation of limited healthcare resources [19, 20].

Qualitative interviews allow the identification of aspects that standardized, predominantly quantitative methods fail to capture - thereby enabling a more nuanced exploration of patients’ qualitative experiences. Moreover, they offer higher flexibility, actively engage patients, and support participatory research methodologies [21]. We may therefore assume that through a subject-oriented approach, respondents are more likely to perceive themselves as partners in the research process, which facilitates greater openness and depth in their responses [22].

A complementary approach to capturing patients’ perspectives beyond predefined frameworks involves qualitative interview analysis as an additional method, alongside specialized forms of PROMs as a practical solution. The use of qualitative interviews and grounded theory can uncover aspects not yet identified or addressed by PROMs, which typically focus on standardized domains without allowing for supplementary input.

To date, few studies in clinical oncology have integrated mixed methods approaches to define treatment goals individually. In 2020 a systematic review of the existing qualitative literature on patient-centred care concluded that effective patient-centred care in oncology requires further systematic assessment of individual needs, guided by patients’ personal values and preferences [12]. Therefore, the aim of this study is to identify potential additional needs of a cohort of gynaecological oncology patients that may be overlooked when relying solely on PROMs, by applying a mixed methods approach incorporating semi-structured qualitative interviews. The primary objective was to reconstruct patients’ values, goals, and psychosocial experiences during cancer treatment. As a secondary analytic step, we mapped interview-derived categories to domains covered by commonly used PROMs to explore alignment and identify potential gaps between standardized measurement domains and narrative experience.

## Methods

### Study design and research approach

This qualitative sub-study is embedded within a broader research project funded by the German Cancer Aid Foundation (Deutsche Krebshilfe), which aims to reconstruct patients’ personal values, therapeutic goals, and their expectations - both positive and negative - regarding social participation, identity, and self-concept during cancer treatment.

The study was prospectively registered with both the Research registry (Registeration ID: 6615128527f813002936e32b) and the German Clinical Trials Register (DRKS 00033242). Ethics approval was obtained from the Ethics Committee of the State Medical Association of Rhineland-Palatinate, Germany (approval no. 2022–16670, version 4.0, dated 23 November 2022, decision issued on 14 September 2022). The trial was conducted in full accordance with the Declaration of Helsinki and relevant national data protection regulations (DSGVO).

A prospective clinical trial employing a mixed-methods approach, integrating qualitative and empirical research methodologies was used. This approach enabled a contextualized, process-oriented analysis of patients’ desires and expression of values over the course of their cancer journey. The qualitative component was guided by constructivist grounded theory [23], which was selected because the study aims not only to describe experiences but to develop conceptually grounded insights into how patients narrate needs, values, and psychosocial meaning during therapy and how these narratives align, or do not align, with the fixed architecture of routinely used PROMs. Grounded theory is suited to this aim because it supports inductive category development closely anchored in participants’ accounts while allowing theoretical integration through constant comparison and memo-writing. The study was conducted between October 2022 and April 2025 at a university medical centre.

### Recruitment and participants

Participants were recruited from affiliated oncology departments at the point of therapy initiation, transition into or during maintenance therapy, following the finalization of individualized treatment plans by the respective interdisciplinary tumor boards. Recruitment took place once the therapeutic regimen had been formally confirmed and prior to the baseline research visit. All participants provided written informed consent after receiving detailed information about the study’s objectives, procedures, and their rights as research participants. They were explicitly informed of their right to withdraw from the study at any time without providing a reason and without any consequences for their ongoing medical care. The study was conducted in accordance with the European General Data Protection Regulation (GDPR/DSGVO) and institutional ethical standards.

A total of 20 participants were enrolled, all of whom had received a histologically confirmed diagnosis of a gynaecologic malignancy and were either preparing to initiate therapy, to change therapy regimen or actively undergoing maintenance therapy. As the study design was based on in-depth interviews that were transcribed and analysed using qualitative methods and grounded theory, the sample size was considered sufficient to generate theoretically grounded and meaningful findings. The generation of data and data analysis was evaluated in a process of statistical counselling. Each participant was interviewed using a semi-structured interview guide that was developed based on a framework derived from existing literature but the structure was applied flexibly to accommodate the unique clinical and emotional circumstances of each participant.

### Eligibility criteria

Participants were eligible for inclusion if they were adult women (>18 years) with a histologically confirmed diagnosis of gynecologic cancer (ovarian, cervical, uterine) or breast cancer, had full decisional capacity, and were able to provide informed consent. Exclusion criteria included current treatment for a psychiatric disorder or cognitive impairment limiting meaningful participation, and advanced age where treatment decisions were primarily guided by geriatric oncology assessments and age-specific therapeutic goals.

### Data collection

Data was collected through semi-structured, in-depth interviews guided by a thematically structured interview framework developed specifically for this study (Supplementary file 1). The guide encompassed three major domains: 1) bodily and physical experiences of illness, 2) individual therapy goals and treatment-related decision-making, and 3) expectations - both positive and negative - regarding social participation, identity, and self-concept. The interview structure was designed to facilitate narrative exploration, with open-ended questions and flexible prompts tailored to the participant’s clinical and emotional context.

All interviews were conducted in person by the lead interviewer, a clinician-researcher and board-certified gynaecologist. Her clinical background supported rapport and enabled discussion of sensitive issues such as side effects, sexuality, fertility, and long-term health priorities. To reduce potential power dynamics, recruitment was undertaken only after treatment plans had been finalized by the interdisciplinary tumor board, and participants were explicitly informed that participation or non-participation would have no influence on their medical care.

Each interview was audio-recorded using a digital recording device, with written informed consent obtained in advance. The recordings were transcribed verbatim and pseudonymized during the transcription process to ensure participant confidentiality. Participants are referenced throughout using neutral identifiers, and sample characteristics are provided in Table 3 to support interpretation of quotations without compromising anonymity. In addition to the recordings, field notes were documented after each interview to capture non-verbal cues, emotional responses, and contextual details that might inform later stages of analysis.

Quotations are attributed to pseudonymized participant codes (e.g., Gyn_8), where “Gyn” indicates a participant recruited in gynecologic oncology and the number represents the internal participant sequence; this format is retained throughout to ensure consistent linkage of quotations across the Results and Tables while maintaining confidentiality.

It should also be noted that, PROMs were not discussed during interviews and the interview guide did not include items about PROM familiarity, acceptability, or perceived usefulness; PROM-related analysis was conducted only as a secondary mapping step after inductive category development.

### Data analysis

The interview transcripts were analysed using principles of grounded theory methodology, following the iterative and comparative logic outlined by Charmaz et al. 2017 [23]. This approach enabled the development of categories and conceptual insights directly rooted in participants’ narratives, with a particular focus on their values, personal treatment goals, and psychosocial impacts of cancer and therapy.

#### *Data management and coding stages*.

Transcripts were uploaded and coded using MAXQDA Analytics Pro (VERBI Software, 2022). The analysis proceeded in three stages: (1) initial (open) coding, where transcripts were read line-by-line and coded inductively to capture participants’ language, metaphors and expressed meanings; (2) focused coding, in which the most significant and frequently occurring initial codes were refined into more conceptual categories; and (3) theoretical coding, where relationships between categories were examined to construct higher-order themes and integrative theoretical insights. Throughout the analytic process, constant comparison was employed; codes and emerging categories were compared across cases and therapy phases to identify variations, patterns, and underlying structures. Analytic memos were used to document interpretive decisions, track category evolution, and reflect on positionality. To increase transparency of the analytic process, examples illustrating the progression from interview excerpts to initial codes, conceptual interpretations, and higher-order thematic domains are provided in Supplementary File 4.

#### Analysis team and collaborative coding

Coding and category development were conducted by an interdisciplinary team including clinical gynecology and ethics/philosophy of medicine. Initial coding was led by the primary analyst, and two additional team members independently coded a defined subset of transcripts to calibrate code meanings and boundaries. Collaborative coding was achieved through regular interpretation meetings in which coding discrepancies were discussed, code definitions refined, and decisions documented, resulting in an audit trail of analytic choices. This consensus-oriented approach was used to enhance reflexivity and conceptual clarity rather than to maximize statistical inter-coder agreement [24, 25].

#### Analytic cadence and saturation

Data collection and analysis proceeded iteratively. Initial coding began shortly after each interview, allowing emerging concepts to inform subsequent interviewing and probing within the semi-structured format. Analysis continued until theoretical saturation was reached- defined pragmatically as the point at which additional interviews no longer yielded substantively new categories or category properties, and subsequent interviews primarily elaborated existing categories rather than altering the category structure.

#### Secondary PROM-domain mapping

For this subset analysis, known and frequently used PROMs in gynecologic and breast oncology were analysed to identify main categories covered by them (Table 1). Five main PROM categories emerged: 1) physical functioning and symptoms, 2) body image and self-perception, 3) psychological wellbeing, 4) social functioning and 5) sexual health. Importantly, these PROM categories were not used as an a priori coding framework for interview analysis. Instead, after the inductive grounded theory category structure had stabilized, we mapped interview-derived categories onto PROM domains to examine alignment and to identify narrative elements that remained insufficiently represented within standardized PROM architectures (Table 2).

**Table 1:** Gynaecologic cancer- specific PROMs including breast cancer

PROM	Focus
EORTC QLQ-OV28 [26]	Ovarian cancer: GI symptoms, chemo side effects, body image, sexual function
EORTC QLQ-CX24 [27]	Cervical cancer: body image, sexual worry/function, lymphedema, treatment symptoms
EORTC QLQ-EN24 [28]	Endometrial cancer: urinary symptoms, lymphedema, body image, sexual health
EORTC QLQ-BR23 [29]	Breast cancer: body image, sexual functioning, systemic therapy side effects, breast and arm symptoms
FACT-O [30]	Ovarian cancer-specific: abdominal discomfort, treatment effects, sexual concerns
FACT-Cx [31]	Cervical cancer: sexual functioning, vaginal issues, body image, treatment side effects
FACT-B [32]	FACT-G + breast cancer-specific concerns: arm morbidity, body image, sexuality, and treatment side effects
BREAST-Q [33]	Satisfaction and QoL after breast surgery: psychosocial, sexual well-being, and physical well-being
PROMIS® tools [34]	Flexible, domain-specific PROMs: fatigue, depression, pain, sexual health (adaptive format)
HADS (Hospital Anxiety and Depression Scale) [35]	Anxiety and depression symptoms, frequently used in breast cancer populations
Body Image Scale (BIS) [36]	Body image disturbances related to cancer and its treatment
Female Sexual Distress Scale (FSDS) [37]	Sexual distress and emotional burden related to sexual dysfunction
FSFI (Female Sexual Function Index) [38]	Sexual function: desire, arousal, lubrication, orgasm, satisfaction, pain

**Table 2:** Analysis framework of identified categories in PROMs and their representaitons

Main category covered by PROM	Included subcategories in PROM	Coding as respresented in interview data	Subcategories in Interview Data	Number	Examples
Physical functioning and symptoms	Fatigue	Body impact	Bodily impact due to illness	32	Due to the metastases, I can no longer hear anything on one side and I had a loss of balance. (Gyn_7)
	Exhaustion	individual limitations	Bodily impact due to sense of having cancer	14	[…] now that I knew it was cancer, you often feel a tugging sensation up there or something […]. (Gyn_9)
			Side-effects of therapy	63	You really notice that you’re actually working against physical decay […] You can definitely tell that- uh- that you have a poison in your body that just doesn’t belong there. You really feel it. (Gyn_2)
			Exposure to side-effects of therapy	52	My legs are quite swollen. I’ve never had that. (Gyn_3)
	Pain		Limitations due to illness itself	6	I’m limited when it comes to exercise. I mean, I actually love working out. But right now, it just doesn’t work. I simply don’t have the strength, or I start exercising and then I just feel nauseous. (Gyn_8)
	Gastrointestinal symptoms		Limitations due to therapy or side effects of therapy	18	Actually being confined to bed like that, that was something I had never experienced before. I used to be a very active person. (Gyn_6)
	Limitations in daily activity		Long-term consequences of therapy	11	I’m a bit scared of these polyneuropathies. And just in general, of whatever else might still show up. Because right now, I simply don’t know. (Gyn_8)
Body image and self- perception	External changes	Body image, Self-image	Change of body image due to illness	17	That does something to your self-image. Because I also defined myself very, very much through my hair. Long, blonde, straight. It suits me. (Gyn_5)
	Shame	Emotional reaction Shame	Change of self-image as a woman due to illness	19	When I lost my hair, I thought, now I’m no longer a real woman. It was mainly about the hair. You cannot see the breast changes if you do not know. Hair was much, much worse for me. (Gyn_7)
	Feminity	Change of self-image as a woman due to illness	Self-image	20	[…] but it still doesn’t change my feeling of being a fully-fledged woman […] I still really feel like a woman in my partnership. (Gyn_5)
Psychological wellbeing	Anxiety, worries	Emotional reactions, mental coping strategy	Hope	18	Even if you have a negative attitude, try to pull yourself into a positive area. My water glass is half full and not half empty. (Gyn_10)
	Depression		Gratitude	16	I also have amazing support from my family- my daughters and their partners or spouses. And the same goes for my friends. No one has fallen away, and that’s always really, really heartening. (Gyn_10)
			Trust	9	Trust is key, and it has to be built. (Gyn_15)
			Guilt	5	The doctor told me that if I had gone to the doctor earlier, if I had caught it sooner, the metastases might not have spread. (Gyn_14)
	Resilience, coping		Sadness	12	I cry all the time […]. I don’t recognize myself anymore. (Gyn_1)
	Sleep problems		Anger	6	It’s just frustrating. The therapy has been going on for almost a year and a half now, always with some kind of treatment, and I’m just really fed up. Honestly, I’m just really annoyed. I can’t put it any other way. […] so I can get back to feeling fit and do things with my kids, who have had to sacrifice a lot over the past year and a half. Yeah. I’m just really fed up. (Gyn_12)
			Fear	59	It was pretty intense. And then the fear started (crying). The fear of dying, of falling apart, just began (crying). (Gyn_1)
			Disappointment	26	I found it really hard to get so little support from my oncologist, who would just keep saying: ‘That doesn’t help us right now either, I just have to push you through this.’ It really left me feeling incredibly alone. (Gyn_5)
Social functioning	Family, partner	Social participation, Emotional reactions	Social resources for coping	50	My husband and I went through this together. Without him, I would have been lost. (Gyn_9)
	Friends		Reduction of social participation	10	Friends who are no longer friends. New ones have been added. (Gyn_12)
	Social isolation		Restriction of social participation due to illness	15	The people around me don’t know how to deal with it. They pull away; they simply can’t cope with the situation. (Gyn_11)
			Reaction of the environment	69	Overwhelming in a positive way- yes, the support. The understanding for the exceptional situation. The offers of help. Just taking me out for a walk, saying, ‘Come on, let’s go for a bit.’ Or, as I said, simply giving me total freedom. (Gyn_5)
			Concerns of reduction of social participation	19	I’m also avoiding parts of my friend group, not because I can’t go due to physical reasons, but because I’m afraid of how people might react. (Gyn_6)
			Perceived feeling in company	14	But a lot of the time, when I go out somewhere, I feel like everyone’s staring at me. […] I looked at myself and thought, I look so different now. And then I just don’t feel like putting myself out there or showing myself to others. (Gyn_6)
Sexual health	Libido	Influence illness on sexuality, change of body image as a woman,	Influence illness on sexuality	9	But I think it’s definitely having an effect […] you certainly have a very restrained libido. (Gyn_13)
		social participation: reaction of environment			
	Partnership		Change of body image as a woman	19	I also don’t feel attractive anymore or anything like that. I’m not in a relationship, and that’s another thing. I used to date before. How will that be now? I have no idea. I just don’t know. (Gyn_6)
	Pain during intercourse		Social participation reaction of environment	14	My husband never hugged me at first. I had the feeling that maybe he was disgusted by me or by the cancer. (Gyn_19)

#### Reporting transparency

This qualitative study is reported in accordance with the COREQ (Consolidated Criteria for Reporting Qualitative Research) checklist; the completed checklist is provided as supplementary file 3.

Credibility was strengthened through iterative analysis alongside data collection, constant comparison across cases, and interdisciplinary interpretation meetings that challenged emerging interpretations. Dependability and confirmability were supported through memo-writing, documentation of coding decisions and category refinement, and the maintenance of an audit trail within MAXQDA. Transferability is supported by transparent reporting of the study context and a structured summary of sample characteristics (Table 3), allowing readers to assess applicability to other settings. Reflexivity was addressed by documenting the interviewer’s dual clinical/research role through field notes and by incorporating interdisciplinary perspectives during analysis to mitigate single-perspective interpretation.

**Table 3:** Sample characteristics

Characteristic	n (%)
Total participants	20 (100)
Age (years), range	31–72
Age (years), mean	49.4
Marital status	
Married/partnered	13 (65)
Divorced	2 (10)
Single	5 (25)
Children	
Has children	12 (60)
Mean number of children (range)	1.3 (1–4)
Education	
High school diploma	4 (20)
Intermediate school-leaving certificate	8 (40)
University level	6 (30)
Primary school or technical college	1 (5)
Therapy phase	
Initiation phase	10 (50)
Transition/maintenance therapy	7 (35)
Escalation due to progression	3 (15)

#### Researcher positionality

The primary interviewer was a clinician-researcher and board-certified gynaecologist working at the participating university hospital. This clinical background facilitated rapport and enabled discussion of sensitive issues such as treatment side effects, sexuality, fertility, and long-term treatment priorities. At the same time, the dual role as clinician and researcher may have influenced the framing of questions and the interpretation of participants’ narratives. In addition, the analytic process involved an interdisciplinary research team including clinical gynecology and medical ethics.

## Results

Twenty women (31–72 years; mean age 49.4) participated. The qualitative analysis yielded 952 initial codes, which were condensed through focused coding into 11 overarching categories. These categories captured how participants narrated treatment experiences in relation to bodily changes, identity, coping strategies, and relational dynamics. The categories were subsequently organized into five higher-order domains, which informed the PROM-aligned framework and the secondary PROM-domain mapping presented above (Table 2).

### Physical functioning and symptoms

As shown in Table 2, the first PROM domain, physical functioning and symptoms, is represented by the subcategories fatigue/exhaustion, pain, gastro-intestinal symptoms and limitations in daily activity.

In our interview data these are subdivided into seven sub-categories: bodily impact due to illness, bodily impact due to sense of having cancer, side-effects of therapy, exposure to side-effects of therapy, limitations due to illness itself, limitations due to therapy or side effects of therapy and long-term consequences of therapy.

Participants mainly expressed side-effects of their therapy that ranged from light to severe manifestations. Questions about side effects of the therapy as well as the process of dealing with them were answered detailed and vividly. Mostly mentioned were loss of power and fatigue followed by flavor changes, joint and bone pain, hair loss, and mucosal dryness. Those who experienced weakness due to therapy described adapting daily routines by planning additional rest periods and distributing tasks over longer time frames. This was often narrated not only as reduced capacity, but as a disruption of valued activities and identity (e.g., sports and physical autonomy):

I just realize that I’m limited, at least when it comes to sports. I actually love sport. But it’s not working at the moment, I don’t have the strength, or I start doing sport and then I just feel sick. (Gyn_8)

While side effects were described vividly and in detail, participants were often more reserved when discussing cancer-related bodily sensations or long-term consequences, suggesting that “symptoms” were not always easily separated from broader meaning-making processes about illness and future self. Most participants responded evasively when asked about disease-related symptoms, personal limitations, physical sensations associated with cancer, or concerns regarding long-term consequences. Patients reported several restrictions due to related side effects of the therapy. These included a reduced ability to engage in sports, the need to limit social contacts to avoid infections, and the logistical burden of frequent hospital appointments, which can disrupt daily routines. Concerns about the long-term consequences of therapy varied among individuals. Some feared a lasting reduction in physical capacity, particularly due to medications that place stress on the heart. Others worried that their hair might not grow back after treatment, or that reconstructive procedures such as breast augmentation with implants might lead to unsatisfactory aesthetic outcomes.

### Body image and self- perception

The second category body image and self-perception is represented by subcategories external changes, shame and femininity. In our interview data these are subdivided into four sub-categories: change of body image due to illness and change of self-image as a woman due to illness, self-image and emotional reaction shame. Overall, respondents emphasized that having as much of the tumour or affected organ removed as possible (regardless of whether it involved breast or abdominal cancer) was very important to them. When explicitly asked whether the illness or surgery had altered their perception of femininity, most participants denied such a change, as well as any significant impact on their partnerships. One participant stated,

[…] but it still doesn’t change my feeling of being a fully-fledged woman […] I still really feel like a woman in my partnership. (Gyn_5)

Across interviews, “femininity” was rarely described as globally diminished; instead, participants differentiated between stable core identity and specific visible changes that altered social self-presentation. This distinction became most evident in narratives about hair loss and scarring, where participants described a mismatch between internal continuity and external appearance. However, women without a current partnership expressed concerns about how potential partners might react to physical changes such as scars, asymmetrical breasts or sagging skin. The loss of hair was repeatedly mentioned as a particularly drastic change, both in terms of body image and in perception of femininity:

When I lost my hair, I thought, now I’m no longer a real woman. It was mainly about the hair. You cannot see the breast changes if you do not know. Hair was much, much worse for me. (Gyn_7)

Despite these changes, participants did not express feelings of shame regarding their bodies.

Social roles within friendship networks were also perceived to have shifted. Participants reported a loss of former positions as initiators and organizers in their social lives. As one participant explained:

Before, I was someone who kept circles of friends together. I am a person who likes many people in one place. I have always organised it that way and now that is completely gone. And you can tell that I am no longer the one who says: ‘So, we’re going there today.’ (Gyn_6)

In addition to bodily changes, participants described shifts in social positioning and roles, indicating that self-perception was strongly mediated through participation and interpersonal function rather than only through appearance. Interestingly, some participants reported not initially identifying as “ill.” It was only through contact with fellow patients or moments of self-recognition - such as catching a glimpse in the mirror - that they were forced to confront their altered self-perception. This internal shift is illustrated in the following reflections:

I think my self-image was different. I was often frightened when I suddenly saw myself in mirrors. You have a completely different image of yourself. […] That is when I realised that I had a different mind-set. I faded that out. (Gyn_17)

Several participants described a delayed integration of the “patient” identity, often triggered by social comparison or moments of self-recognition, highlighting that identity disruption emerged as a process rather than a single event. For some, the notion of a “new self” was met with resistance. One participant explicitly rejected the idea of transformation:

Many say: ‘That will be a new you.’ Yes, but I never wanted a new me. I actually always wanted to be the way I am. (Gyn_6)

Conversely, other participants emphasized that their self-image had remained largely intact. They underscored the continuity of core personality traits, values, and inner strength, despite the illness:

Giving up is not an option for me anyway. (Gyn_7)

Taken together, these accounts show that changes in self-perception ranged from disruption and resistance to reaffirmation of identity, with visible bodily changes and altered social roles acting as key drivers of how participants narrated “self” during treatment.

### Psychological wellbeing

The third main category is psychological wellbeing including mental coping and emotional reactions under subcategories of hope, gratitude, trust, guilt, sadness, anger, fear and disappointment. Across interviews, participants described psychological coping as an active and ongoing process shaped by therapy phase, perceived control, and relational security. Two overarching patterns were prominent: (1) the effort to maintain hope and normality as protective resources, and (2) the management of vulnerability through selective engagement with support and illness-related communication. Mental coping strategies emerged as a central.

Many participants emphasized the need to preserve hope and optimism- even in constrained or uncertain prognostic situations- as a prerequisite for psychological stability. Hope was often described in pragmatic terms as something to hold on to when facing treatment demands and ambiguity. One participant captured this need for a minimal but sustaining orientation toward the future, “I need a straw to hold on to.” (Gyn_1)

Acceptance and reframing were repeatedly described as strategies to regulate distress. Several participants articulated a deliberate attempt to shift perspective and to interpret their situation in a way that enabled endurance and everyday functioning. As one participant stated: “Even if you have a negative attitude, try to pull yourself into a positive area. My water glass is half full and not half empty.” (Gyn_10)

At the same time, tension between vulnerability and control was evident. Psycho-oncological support and self-help groups were generally perceived as potentially valuable, yet they were often declined or postponed. Reasons included a strong sense of self-sufficiency ambivalence about whether external support would be helpful, and a desire to avoid being positioned primarily as a “patient.” This ambivalence was reflected in retrospective self-questioning:

Maybe I made a mistake. I had been given a card from a psychologist … I did not call. I muddled through on my own. (Gyn_11)

A closely related coping strategy was the attempt to preserve normality. Participants described efforts to maintain familiar routines and social roles, as well as practices that supported continuity of self-presentation (e.g., cosmetics, wigs, everyday structure). Normality was framed not as denial, but as a stabilizing resource that reduced the psychological salience of illness: “The most important thing is normality. To lead as normal a life as possible.” (Gyn_10)

In addition, participants frequently engaged in selective communication to regulate emotional exposure. Many limited conversations about illness to avoid being repeatedly confronted with their diagnosis or reduced to a patient identity. This included boundaries toward family, friends, and broader social environments, particularly when participants experienced such conversations as emotionally exhausting or as preventing psychological recovery. One participant expressed this desire for distance from the illness narrative directly: “I’d prefer to just forget that I’ve been diagnosed with cancer.” (Gyn_9)

Disappointment was frequently tied to unmet expectations, both within health care system and in personal relationships. Participants described distress when communication was unclear, when they perceived gaps in reliability of care, or when social support felt inconsistent. Such experiences contributed to emotional insecurity and diminished trust. Interpersonally, disappointment was described when participants felt emotionally neglected or burdened by managing others’ reactions.

Emotions such as guilt, sadness, and anger were often intertwined. Guilt was expressed as concern about burdening family members or regret about delayed symptom recognition. Sadness was associated with multiple forms of loss, including physical function, reduced social participation, and a perceived distance from one’s former identity. Anger was typically directed toward systemic issues such as delayed treatments, fragmented coordination, and unsatisfactory communication. Across these emotional responses, participants described periods of emotional instability, withdrawal, and a heightened sensitivity to perceived loss of control.

Despite distress, gratitude and hope appeared as counterweights and were frequently anchored in supportive relationships and moments of reliable care. Gratitude was directed toward both informal networks (partners, family, friends) and formal support (healthcare providers), particularly when participants experienced communication as transparent and supportive. Trust in healthcare professionals emerged as a key emotional concern and was closely linked to honesty, clarity, and reliability in interactions; when these were present, participants reported greater emotional containment, whereas perceived inconsistencies amplified fear and disappointment.

Taken together, psychological wellbeing was shaped by an ongoing negotiation between maintaining hope and normality, protecting autonomy through selective engagement, and responding to fluctuating emotional states across the treatment trajectory.

### Social functioning

The fourth main category social functioning is represented through six subcategories in the interview data including social resources for coping, reduction of social participation, restriction of social participation due to illness, reaction of the environment, concerns of reduction of social participation and perceived feeling in company.

Participants reported significant reductions in social participation due to a combination of physical, psychological, and contextual factors. Physical limitations including fatigue, pain and reduced mobility were major barriers to engage in social activities. Psychological challenges, such as anxiety, low mood or internal resistance further diminished social engagement. Heightened infection risk during immunocompromised periods (e.g., chemotherapy) prompted deliberate avoidance of crowds and enclosed spaces, resulting in behavioural adaptations like attending public places during off-peak hours. Concerns about infections like COVID-19 and influenza increased isolation and disrupted social routines. Although digital and telephone communication served as partial substitutes, many participants emphasized their emotional limitations and experienced feelings of social loss and disconnection, particularly those formerly active in community or volunteer roles.

Reactions from the social environment varied by relationship and circumstance reflecting significant variability in emotional and practical support. Some noticed no changes in others’ behaviour while others experienced diverse responses. In intimate partnerships, reactions varied notably. Some spouses offered caring and non-verbal support, such as physical comfort, while others initially displayed distancing behaviours causing emotional hurt.

My husband never hugged me at first. I had the feeling that maybe he was disgusted by me or by the cancer. (Gyn_19)

In family dynamics shifts varied by life stage. Families with young children struggled with everyday organization and financial uncertainty, whereas participants with adult children were more concerned about the emotional impact on their ageing parents.

You are not just ill yourself; everyone around you suffers, too. Maybe the relatives suffer even more than you do sometimes. […] so we didn’t know if we can handle the financial situation. (Gyn_12)

In friendships, experiences ranged from increased closeness and support to superficiality or distancing due to friends’ uncertainty or fear of causing offense. Several participants overwhelmed by attention or obliged to regularly update friends to avoid worry. Unexpected support emerged from previously distant acquaintances or workplace colleagues, who provided emotional support and financial assistance, helping to stabilize participants’ sense of normality. In response to this participants reported commonly a reprioritization of relationships, illustrated by the loss of some friendships and the formation of new ones:

Friends who are no longer friends. New ones have been added. (Gyn_12)

Concerns about reduced social participation initially centred on fears related to negative body image and being perceived differently. Over time, however, these fears appeared to diminish, especially when participants experienced stability and support from close relationships that acted as protective factors maintaining social connectedness. Long-term social exclusion was not a primary concern; participants expressed optimism about resuming social activities following physical rehabilitation. Younger participants briefly noted potential future challenges such as family planning but generally avoided engaging deeply with these issues during illness.

Social participation and available social resources were crucial for coping. Participants reported receiving intensive emotional and practical support primarily from spouses or partners, who played a vital role in caregiving and shared coping:

My husband and I went through this together. Without him, I would have been lost. (Gyn_9)

Adult children contributed in organizational and emotional assistance, sometimes with professional expertise, while grandchildren provided emotional comfort. Parents and siblings mainly supported with daily living tasks and offered emotional presence. Close friends were essential sources of support through listening, accompanying to appointments, and spontaneous acts of kindness. Long-standing friendships, particularly those with a shared illness experience, were especially valued. Neighbours contributed practical help and low-threshold everyday support. Many participants noted that “Some relationships have become closer or perhaps even more honest.” (Gyn_13)

Professional social support from colleagues and supervisors was perceived as valuable, with some workplaces facilitating emotional and practical support that helped maintain a sense of normality. Engagement with community groups such as sports clubs, volunteer organizations, or religious groups was consciously maintained or sought, to preserve structure, enjoyment, and emotional strength. Psychological support was recognized as helpful and utilized when needed, with empathetic healthcare providers playing a key role in emotional support.

These variations suggest that social functioning is shaped less by the mere presence of a network and more by the predictability, reciprocity, and emotional safety of relationships during periods of vulnerability.

### Sexual health

As previously outlined in 3.2, there were hardly any effects on the respondents’ perception of being a woman. Most participants in partnerships stated a more reserved sexual activity compared to the time before the illness. This change was primarily attributed to reduced libido due to physical limitations such as side effects of the therapy including fatigue; concerns that sexual activity could have a negative impact on the physical healing process and mental distraction due to illness. Nevertheless, it was emphasized that the relationship itself remained intimate, sustainable and in some cases led to an even closer bond. Overall, the topic was not very present for the participants. Sexuality was rarely foregrounded spontaneously; when discussed, participants framed changes primarily as temporary consequences of fatigue, reduced libido, and mental preoccupation, while emphasizing continuity of intimacy and partnership stability.

The side effects are certainly what is primary at the moment. But it’s also getting to know your body again somehow. And I don’t think it will be like this forever. I think it’s just a phase. And I also believe […] that we’ll manage it again. But I think it’s definitely having an effect […] you certainly have a very restrained libido. (Gyn_13)

## Discussion

PROMs are widely used tools for capturing patient perspectives in oncologic care [39–41]. Their integration into gynaecologic oncology has advanced standardization and accountability by systematically quantifying physical symptoms, psychological wellbeing, social participation, body image, and sexual health. However, our findings align with a growing body of literature indicating that while PROMs are highly patient-reported, they are not inherently patient-centred [42–45].

This study demonstrates that although PROMs encompass relevant domains, they frequently fall short in granularity, contextual sensitivity, and adaptability to individual meaning-making. The mixed-methods approach employed here - combining PROM domain analysis with in-depth qualitative interviews- uncovered experiential dimensions that remain insufficiently represented within the fixed architecture of standard PROMs. This includes nuanced expressions of body image, social role renegotiation, coping strategies, and the ambivalence surrounding psycho-oncological support.

First, the PROM domains we analyzed (physical functioning, psychological wellbeing, social and sexual functioning, and body image) are indeed salient; participants spontaneously addressed all of them. Yet the ways in which these topics were narrated reveal the limitations of structured instruments. PROMs excel in identifying the presence of a symptom but not its subjective weight. For example, hair loss - already acknowledged in some PROMs - was described by participants not simply as a cosmetic concern, but as a profound symbol of identity disruption and social visibility. Conversely, hair regrowth emerged as a new source of tension, as it falsely signaled recovery to others, obscuring ongoing struggles. This complexity escapes current PROM structures.

Similarly, participants described fatigue not only as a physical state, but as a disruption to social identity and role fulfillment. Attempts to maintain normality, preserve autonomy, or resist emotional vulnerability emerged as central coping strategies, none of which are adequately captured by PROM checklists. These findings underscore that symptom checklists often fail to elucidate the underlying existential or relational significance of patient experiences.

Second, PROMs often function under the assumption that symptom burden equates to a desire for intervention. However, our data suggest that some participants actively resisted engagement with psycho-oncological services, despite acknowledging distress. This distinction, between identified needs and negotiated needs, highlights a critical limitation of PROMs: they may over-pathologize experiences or overlook the patient’s agency in meaningfully declining certain forms of support. The nuance required to interpret such ambivalence cannot be derived from quantitative scoring alone.

Moreover, emotional regulation strategies such as selective disclosure, distancing from distressing topics, and the preservation of everyday routines functioned as protective mechanisms. PROMs typically assess symptom severity but do not illuminate these adaptive responses, nor do they account for when non-engagement reflects considered agency rather than unmet need.

Thirdly, social functioning is formally addressed in many PROMs, yet our findings indicate that its actual expression is far more layered. Participants reported dynamic changes in social identity, interpersonal roles, and social network composition. These included both alienation and unexpected solidarity, reprioritization of relationships, and the emotional complexity of being perceived by others as “ill” or “recovered.” Such dimensions, shaped by relational context and life history, elude categorical documentation but are crucial for understanding patient wellbeing and long-term psychosocial integration.

Finally, the domain of sexual health was addressed less frequently in our interviews, and when it was, it emerged in relational rather than functional terms. Participants emphasized emotional closeness and partner communication over specific sexual activity or dysfunction. Here, PROMs may serve a valuable role in introducing topics that patients may otherwise avoid. However, when interpreted in isolation, they risk medicalizing silence or misattributing significance to numerical scores, divorced from the relational and temporal context in which sexuality is negotiated.

In this study, the qualitative findings and the PROM-domain comparison indicate that commonly used PROM structures cover relevant domains, yet do not consistently capture the depth, contextual meaning, and relational/identity-related dimensions that were salient in participants’ accounts. On this basis, we outline the following cautious implications for PROM interpretation and future instrument development. These points are offered as practice-oriented considerations arising from our data and as hypotheses for future evaluation; they were not directly tested as implementation interventions within the present study.

First, PROM use could be complemented by brief open-ended prompts (for example, one or two short questions) to allow patients to explain what a rating means in their situation and to raise concerns not represented by fixed response options. Second, themes that were repeatedly meaningful in narratives but were not consistently represented within standard PROM architectures- such as hair-related concerns (including regrowth), identity-related uncertainty, or relationship dynamics and resilience- may warrant consideration for additional items or optional add-on modules. Any such adaptations would require careful development, patient involvement, and subsequent psychometric validation. Third, PROM scores may be most useful when interpreted as hypothesis-generating signals that guide follow-up rather than as definitive indicators of unmet need. Our findings suggest that expressions of burden can coexist with preferences to decline or postpone certain forms of support; brief qualitative clarification may therefore be necessary to distinguish unmet needs from informed preferences and to reduce the risk of over- or under-response.

In addition, there are implications for digital PROM systems (ePROMs). Hybrid input formats combining structured scales with optional narrative fields may help preserve contextual meaning while maintaining standardization; however, feasibility, clinician usability, and workflow impact would need to be evaluated empirically before broader implementation. Implementation strategies may also benefit from training clinicians not only in PROM administration but in interpretive competence- how to contextualize PROM outputs, recognize ambiguity, and initiate proportionate, patient-centred dialogue. Finally, future research could examine whether PROM information, particularly when accompanied by brief narrative context, can support shared decision-making and goal-setting in multidisciplinary settings (e.g., tumour boards) and whether it can be integrated into therapy planning and follow-up to better align care with patients’ reported experiences, goals, and functional outcomes over time.

Overall, our study supports the view that PROMs are valuable and scalable tools, but may be incomplete when used as a stand-alone approach to capturing the patient voice. Their strengths lie in standardization and comparability; their limitations become apparent when complex illness experiences require contextual interpretation. In the context of personalized oncology, integrating PROMs with structured opportunities for narrative contextualization could help strengthen patient-centred care and reduce the risk of misinterpretation of patient-reported data.

## Limitations

This single-centre study recruited participants at specific clinical transition points (initiation, transition into/maintenance therapy, escalation due to progression). While this purposive sampling enabled comparison across therapy phases, transferability to other contexts (e.g., different health systems, community settings, or more diverse sociodemographic populations) should be assessed cautiously. The interviewer’s dual role as clinician-researcher likely facilitated rapport and discussion of sensitive issues, but may also have influenced what participants emphasized or withheld. Reflexive field notes, interdisciplinary interpretation meetings, and documentation of analytic decisions were used to mitigate these effects; nevertheless, socially desirable responding and setting-specific narrative framing cannot be fully excluded.

The interviews were designed to elicit patients’ values, goals, and psychosocial experiences and did not include questions about PROM awareness, acceptability, or perceived usefulness. PROMs were not explicitly discussed with participants. Therefore, the PROM-related component of this study should be interpreted as a secondary domain-alignment analysis rather than as an assessment of patient attitudes toward PROMs or an evaluation of PROM implementation. Future studies should directly elicit patient perspectives on PROM use and test whether adding narrative prompts or interpretive follow-up improves clinical utility.

## Conclusion

Taken together, our findings, and the secondary mapping of interview-derived categories to commonly used PROM domains, support the view that PROMs alone are unlikely to be sufficient for delivering consistently patient-centred care. Their potential is most likely to be realized when PROM outputs are embedded within relational, dialogical, and interpretive clinical practices that enable clinicians to clarify meaning, preferences, and goals. Future PROM use in (gynecologic) oncology may therefore benefit from structured opportunities for narrative contextualization and from clinician training that strengthens interpretive competence and reflexivity. Such a mixed-methods orientation may improve the accuracy of need identification, reduce the risk of misinterpretation, and align PROM-informed care more closely with individualized, participatory oncology care.

## Electronic supplementary material

Below is the link to the electronic supplementary material.

Supplementary material 1

## Data Availability

The datasets generated and analysed during the current study are not publicly available due data protection and until final evaluations have been completed. They are available from the corresponding author in psyeudonymized form on reasonable request.

## Abbreviations

PROM: Patient-reported outcome measure
DRKS: Deutsche Register Klinischer Studien
DSGVO: German national data protection regulations
