# Supplementary material for: PROMs: Patient related or patient centred? A bridge between Patient-Reported Outcome Measures (PROMs) and clinical ethics in the context of a cohort of gynaeco-oncological patients analysed by qualitative semi- structured interviews
Source: J Patient Rep Outcomes. 2026 Mar 28;10:55. doi: 10.1186/s41687-026-01050-z (PMC13065924; doi:10.1186/s41687-026-01050-z)
Supplement: Supplementary file 1 — Supplementary material 1 [file 41687_2026_1050_MOESM1_ESM.pdf]

Supplementary File 4. Grounded Theory Analytic Framework and Coding Process

The qualitative analysis followed principles of constructivist grounded theory (Charmaz, 2017). Interview transcripts were coded inductively using a line-by-line approach, generating initial codes that captured participants' language and experiences. Through focused coding and constant comparison across interviews, related codes were grouped into broader conceptual interpretations that informed the thematic domains presented in the Results section of the manuscript.

To enhance transparency of the analytic process, Table S1 illustrates examples of the progression from interview excerpts to initial codes, conceptual interpretations, and higher-order thematic domains.

Table S1. Example of grounded theory analytic progression from interview data to thematic domains

Interview excerpt	Initial code (line-by-line coding)	Focused code / conceptual interpretation	Higher-order thematic domain
"Due to the metastases, I can no longer hear anything on one side and I had a loss of balance." (Gyn_7)	physical limitation due to illness	bodily impact of illness	Physical functioning and symptoms
"You really notice that you're actually working against physical decay… you feel you have a poison in your body." (Gyn_2)	therapy burden	side-effects of treatment	Physical functioning and symptoms
"When I lost my hair, I thought now I'm no longer a real woman." (Gyn_7)	hair loss affecting femininity	altered self-perception	Body image and self-perception
"Even if you have a negative attitude, try to pull yourself into a positive area." (Gyn_10)	positive reframing	coping strategy	Psychological wellbeing
"I cry all the time… I don't recognize myself anymore." (Gyn_1)	emotional distress	sadness / psychological burden	Psychological wellbeing
"My husband and I went through this together. Without him, I would have been lost." (Gyn_9)	partner support	social resources for coping	Social functioning
"Friends who are no longer friends. New ones have been added." (Gyn_12)	changing friendships	restructuring of social network	Social functioning
"You certainly have a very restrained libido." (Gyn_13)	reduced libido	sexuality affected by illness	Sexual health
